# The Mental Deficiency Act

**Published:** 1914-12-05

**Authors:** 


### THE MENTAL DEFICIENCY ACT.

### Difficulties of Administration. Much Work—Small Results.

In The Hospital a fortnight ago we expressed the view that the institutional treatment of lunacy and mental deficiency in London would never be efficient and economical until the London County Council is made the sole authority for its provision and the mental hospitals of the Metropolitan Asylums Board are transferred to it. The difficulties which the London County Council have already experienced in carrying out the provisions of the Mental Deficiency Act afford ample proof that the present position is far from being satisfactory. Since it endeavoured to put the Act into working order the Mental Hospitals Committee have been beset with very considerable difficulties of administration, and they have wisely taken the public into their confidence to explain why the results of their seven months' working are so comparatively small.

It is pointed out that on some points which the Act leaves for definition by regulation, such as the transfer and discharge of patients from institutions, regulations have not been issued even yet.

### The Definition of "Neglect."

It has been found that many cases, of which particulars are communicated in the hope that they will be dealt with, are excluded by the strict limitation that the cases which may be dealt with are only those in which defectiveness has existed from birth or from an early age. Equally difficult has been the task of the committee to define what constitutes "neglect." In certain cases the committee has an obligatory duty to deal only with those who are found neglected, abandoned, or without visible means of support, or cruelly treated. The committee has adopted the principle that the word "neglected" can cover cases where, without wilful omission, the care and accommodation provided, which might be adequate for a normal person, are inadequate for one who is defective. This contention that neglect may be constructive as well as positive has enabled action to be taken in a number of cases where a parent of a defective child, while prepared to give the best care and attention in his power, feels that institution care, which he cannot himself afford, is needed in the child's interests. Difficulty has arisen in some cases owing to the fact that the written consent of the parent or guardian has to be given for the making of an order for detention in an institution (unless the judicial authority considers that the consent is unreasonably withheld). In the first case dealt with under the Act by petition the mother's consent was withheld and the judicial authority, after very careful consideration, felt that he could not pronounce her attitude to be unreasonable. A second petition was then presented to another judicial authority, who took a different view, and an order for the defective to be sent to an institution was ultimately made. Much difficulty has been experienced in some cases in determining as to the liability of the Council or other local authority to take action.

On the difficult question of accommodation it has been felt that until the scope of the Act is somewhat more clearly ascertained it would not be proper for the County Council to embark upon the provision of specially built institutions, but inquiry has been made as to properties which could be taken on lease and adapted at small cost for temporary use. Many properties have been inspected, but few have appeared suitable for use as accommodation for cases of mental defect. It has already been decided, however, to purchase the mission schools at Streatham for use in the first instance for defective females of certain classes, and as a place of safety and receiving home to provide accommodation for some seventy cases. Another property is receiving consideration with a view to a lease, and it is proposed to use the old industrial school at Brentwood for male cases of defect. The only accommodation actually in use so far is what has been provided by means of arrangements with voluntary agencies. Twelve male cases and twelve female are in institutions provided by the Incorporation of National Institutions, and one female case is in St. Mary's Home, Alton. The cases of which information is received from the police are among the most difficult of those with which the Council is expected to deal, and the problem of finding suitable accommodation, in the absence of any provided specially by the Council, is at present acute. Many of them are cases of prostitutes, in which there seems small prospect of moral improvement, and practically all the accommodation for defective prostitutes which is available under contract arrangements is confined to those of comparatively improvable mental type who are susceptible of moral improvement. Negotiations, however, are being pursued for accommodation for this particular class. As regards cases of defectives in prison it appears that the Home Office communicate a great number of cases to the Board of Control, but they are at present refraining from writing to the local authority, as they realise the difficulty occasioned at present by lack of accommodation. The need for providing for this class of case, however, is being urged, and apparently the prisons may be expected to be a source whence many cases subject to be dealt with at the Council's expense will come. Altogether information has been received from various sources of 201 alleged cases of mental neglect, of which ninety-six have been ascertained as subject to be dealt with. Twenty-five cases have been sent to institutions, six have been placed under supervision, and ten sent to places of safety pending the presentation of a petition. No action could be taken in twenty-seven cases, owing to lack of suitable accommodation, five were thought not liable to be dealt with by the London County Council, and forty were found, after inquiry and medical examination, not to be subject to be dealt with under the Act.

